# Sensitive Quantification of Aflatoxin B_1_ in Animal Feeds, Corn Feed Grain, and Yellow Corn Meal Using Immunomagnetic Bead-Based Recovery and Real-Time Immunoquantitative-PCR

**DOI:** 10.3390/toxins6123223

**Published:** 2014-12-02

**Authors:** Dinesh Babu, Peter M. Muriana

**Affiliations:** Department of Animal Science and the Robert M. Kerr Food & Agricultural Products Center, 109 FAPC Building, Monroe Street, Oklahoma State University, Stillwater, OK 74078-6055, USA

**Keywords:** aflatoxin B_1_, immunomagnetic bead capture, real-time immunoquantitative PCR, animal feeds, feed grains

## Abstract

Aflatoxins are considered unavoidable natural mycotoxins encountered in foods, animal feeds, and feed grains. In this study, we demonstrate the application of our recently developed real-time immunoquantitative PCR (RT iq-PCR) assay for sensitive detection and quantification of aflatoxins in poultry feed, two types of dairy feed (1 and 2), horse feed, whole kernel corn feed grains, and retail yellow ground corn meal. Upon testing methanol/water (60:40) extractions of the above samples using competitive direct enzyme linked immunosorbent assay, the aflatoxin content was found to be <20 μg/kg. The RT iq-PCR assay exhibited high antigen hook effect in samples containing aflatoxin levels higher than the quantification limits (0.1–10 μg/kg), addressed by comparing the quantification results of undiluted and diluted extracts. In testing the reliability of the immuno-PCR assay, samples were spiked with 200 μg/kg of aflatoxin B_1_, but the recovery of spiked aflatoxin was found to be poor. Considering the significance of determining trace levels of aflatoxins and their serious implications for animal and human health, the RT iq-PCR method described in this study can be useful for quantifying low natural aflatoxin levels in complex matrices of food or animal feed samples without the requirement of extra sample cleanup.

## 1. Introduction

Animal feedstuffs and agricultural commodities may contribute to the transfer of certain health hazards such as dioxins, mycotoxins, heavy metals, drug residues, pesticides, and microbiological hazards (e.g., *Salmonella*) into the food chain, raising global concerns about food safety. These hazards in animal feeds are usually prioritized based on their relevance to public health, extent of occurrence, and their impact on trade [1]. Mycotoxins are prioritized as ‘unavoidable natural contaminants’ in food and feedstuffs that if consumed may cause serious consequences to animal health [2]. Affecting nearly 25% of the world’s food crops annually [3,4], mycotoxin-related losses in the United States are estimated to range from $0.5 million to over $1.5 billion annually [5], with a mean annual economic cost of $932 million in crop losses [6]. Mycotoxins are produced by several fungi under certain conditions of temperature, excessive moisture, relative humidity, drought, insect damage, variation in crop harvesting practices, and nutrient availability if favorable for the growth of molds [7,8,9,10,11]. Aflatoxins are mainly produced by the *Aspergillus flavus* and *A. parasiticus* fungi and are commonly encountered in foodstuffs and animal feeds worldwide [12,13]. Among the several types of aflatoxins, including B_1_, B_2_, G_1_, G_2_ and M_1_, Aflatoxin B_1_ (AFB1) is the most toxic and prevalent member of the group [14,15,16]. AFB1 can enter a human or animal system through ingestion, inhalation, or dermal contact [6,17], causing a wide range of adverse acute and chronic toxic effects [14,18,19,20,21,22]. In order to avoid ill effects on human and animal health due to frequent occurrence and associated toxicity of aflatoxins, several countries have set maximum permissible limits in commodities of food and feeds. These limits are not universal to all countries. For example, in the United States, the U.S. Food & Drug Administration (FDA) has set the action levels for aflatoxins to be 20 μg/kg for feedstuffs and 0.5 μg/kg for aflatoxin M_1_ (http://www.fda.gov/ICECI/ComplianceManuals/CompliancePolicyGuidanceManual/ucm074703.htm), and in the European Union, the regulatory limits for aflatoxin B_1_ in foodstuffs is at 2 μg/kg and for aflatoxin M_1_, it is at 0.05 μg/kg (http://eur-lex.europa.eu/LexUriServ/LexUriServ.do?uri=OJ:L:2010:050:0008:0012:EN:PDF). Because of the low permissible limits for aflatoxins and the associated high toxicity of aflatoxins impacting health even at sub-chronic exposure levels, the analytical methods for determination of aflatoxins need to be both sensitive and specific to be able to quantify trace levels. Aiming to achieve the safety of foods and foodstuffs and minimize associated regulatory/trade losses, the food and feed industry is in constant pursuit of rapid and reliable methods for detection and quantification of aflatoxins. Among the several available methods for aflatoxin detection, immunoassay methods are proven to provide such assurance during routine diagnostic applications due to the high selectivity and high affinity of antibodies specific for the antigen. Although methods such as radio immunoassays (RIAs), high performance liquid chromatography (HPLC), and enzyme-linked immunosorbent assay (ELISA) have been widely explored for aflatoxin detection, these techniques may require extensive sample cleanup, may take a longer analysis time, and need trained personnel. However, the advantages of immunoassays can be combined with the enormous DNA amplification potential of polymerase chain reaction (PCR), as has recently been done in the immuno-PCR (iPCR) approaches that have become popular for sensitive antigen detection. Boasting a 10–1000-fold increase in limit of detection over the traditional ELISA methods [23,24], immuno-PCR methods allow quantification of an antigen with greater rapidity and sensitivity. Surprisingly, the use of this highly sensitive real-time immuno-PCR approach has not been exploited to quantitatively determine contamination of mycotoxins such as aflatoxin B_1_ in foodstuffs, animal feeds, or feed grains. This could mainly be due to the matrix complexity of these sample types.

Recently, we developed a real-time immunoquantitative PCR (RT-iqPCR) method to detect aflatoxin B_1_ in a methanol/water solvent commonly used for aflatoxin extraction from foods, grains and feedstuffs [25]. Using this method, quantities as low as 0.1 μg/kg of aflatoxin B_1_ were detected, which falls well below the regulatory requirements in the United States and European Union for agricultural commodities. Some of the advantages in quantifying low levels of aflatoxins beyond the detection limits of popular ELISA methods are that one can establish a stricter quality assurance of finished products for better trade and export value, eliminate transfer of toxins in the food chain in commodities such as milk and eggs, and attain accurate diagnoses in the case of chronic toxicity to humans and animals. It was hypothesized that the robustness of the magnetic bead-based qPCR methodology, together with dilution-based extraction schemes to overcome high antigen hook effects, could be adopted to detect and quantify low levels of aflatoxins in natural feed and food samples. The sensitive detection and quantification of aflatoxin in poultry, horse, and dairy animal feeds, whole kernel corn feed grains, and also in yellow ground corn meal used for human consumption were demonstrated in this study. The methodology used here included first subjecting the sample extracts to a USDA-approved competitive displacement ELISA (CD-ELISA) test for initial evaluation of aflatoxin content and then to the RT-iqPCR assay, which is a simplified noncompetitive sandwich immunoquantitative PCR approach for detection and quantification of aflatoxin B_1_ [25]. It is demonstrated that a methanol/water extract of feeds and feed grains can be directly used, without further sample cleanup, for sensitive quantification of aflatoxin levels using our RT-iqPCR assay. Further, the assay is shown to be useful for eliminating false negative samples and the high antigen hook effect commonly encountered in immunoassays.

## 2. Results and Discussion

### 2.1. Estimation of Aflatoxin Using CD-ELISA

Methanol extracts of all samples (whole kernel corn feed grains, dairy feed, poultry feed, horse feed, and yellow ground corn meal) were tested using Neogen’s Agri-Screen test kit (Neogen, Lansing, MI, USA); AFB1 levels were estimated to be less than 20 μg/kg of total aflatoxins as measured visually using color intensity. However, this measurement is semi-quantitative and the actual concentrations of aflatoxin cannot be stated, which is a limitation of this approach; while it can serve as a preliminary rough estimation of the aflatoxin content in the extracted samples, further confirmation for quality assurance may be needed.

### 2.2. Detection and Quantification of Aflatoxin B_1_ Using Real-Time Immune-Quantitative-PCR (RTiqPCR)

The second part of the study involved estimation of aflatoxin content in methanol/water extracts of unspiked and AFB1-spiked animal feeds, whole grain feed corn, and yellow ground corn meal samples using RT-iqPCR. Methanol/water was chosen because it is the commonly used solvent for animal feeds and feed grains for extraction of aflatoxins and the use of water and hydroxylated solvents like methanol is highly recommended to facilitate the effective release of aflatoxins into the extractant solution [26,27]. The analysis of feedstuffs often involves several steps of extraction, filtration, sample cleanup, and concentration before subjecting to mycotoxin assays. Assay sensitivity and the release of bound aflatoxin may be affected by the complexity of food and feed matrices due to the interference of undefined and defined constituents of proteins, fats, and carbohydrates. However, the extraction step used in the method described herein requires no further time-consuming sample cleanup step. Further, the use of antibody-coated magnetic beads as solid support for sandwich immuno-PCR can be conveniently used for manual or semi-automatic bead recovery of mycotoxins using the Dynal bead retriever. The antibodies used in this study were unaffected by methanol solvent, and the toxin precipitation was rapidly done by immobilizing the toxin-captured antibodies onto protein G magnetic beads, as described previously [25].

**Figure 1:**
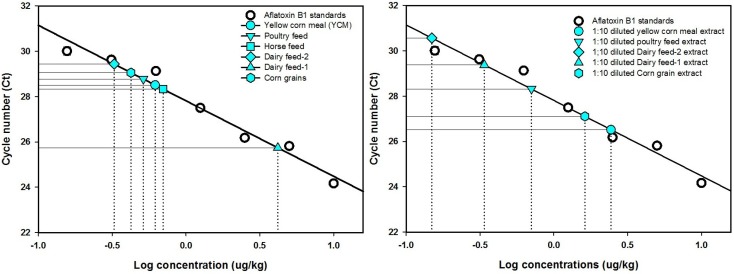
Quantification of aflatoxin B1 in animal feed, food grain, and yellow corn meal extracts against a standard curve. Undiluted (**left**) and diluted feed extracts (**right**) were subjected to double antibody sandwich assay using PureProteome protein G beads. Undiluted feed extracts were compared with 10-fold diluted extracts.

Real-time PCR was optimized (Table 1) to obtain a standard calibration curve, indicating a real-time PCR efficiency of 99.52% with *R*^2^ value of 0.96, slope −3.33, and 27.81 intercept. The detection limits of RT-iqPCR assay for the standard AFB1 quantification was between 0.1 and 10 μg/kg and for concentrations above 10 μg/kg, a high antigen dose hook effect (Prozone) was observed while developing calibration curves (Figure 1). This effect is commonly seen in immunoassays involving antigen concentrations beyond upper detection limits [28,29,30]. If not addressed, the hook effects could potentially exhibit false-negative results, showing higher cycle threshold numbers (*i.e.*, low toxin concentrations) in samples containing high antigen concentrations during the real-time PCR assay. In such cases, the use of sample dilution protocols is suggested [31,32,33] and may require the use of at least four- to ten-fold dilutions of sample extracts to detect the hook effect from samples containing unknown amounts of toxin levels greater than upper detection limits. Therefore, we opted to verify this phenomenon with high aflatoxin concentrations and avoid possible false negatives by performing measurements using undiluted and diluted extracts, as suggested by several researchers who have encountered the hook effect [31,32,33]. Thus, for samples containing unknown amounts of antigen in excess of hook effect levels, initial optimizations should be done to arrive at particular fold dilutions that can be standardized for routine estimations. At first, the immunoassay using 4-fold diluted extracts for corn and yellow corn meal extracts was tried, but the PCR fluorescence plots showed poor distinction between the undiluted and diluted extracts, with very close *Ct* values indicating a need for using more diluted extracts for immuno-capture of aflatoxin. Thus, in order to obtain distinguishable fluorescence signal plots, a 10-fold dilution for sample extracts containing native aflatoxin and 10- and 40-fold dilution for extracts prepared from samples spiked with 200 μg/kg aflatoxin B_1_ was adopted.

**Table 1:** Primer sequences used to amplify amino-modified detector DNA and an internal real-time PCR target sequence.

Primers	Oligonucleotide sequences (5'-3')	Modification	PCR product size	Application of PCR product
pGL2A_Fwd	NH_2_-(C_6_)-GTTCGTCACATCTCATCTAC	5'-amino	560-bp	Tethered via SMCC to anti-AFB1 antibodies
pGL2A_Rev	TCGGGTGTAATCAGAATAGC	None		
pGL2B_Fwd	GAACTGCCTGCGTCAGATTC	None	101-bp	Real-time PCR fragment for quantification
pGL2B_Rev	AACCGTGATGGAATGGAACAAC	None		

**Figure 2:**
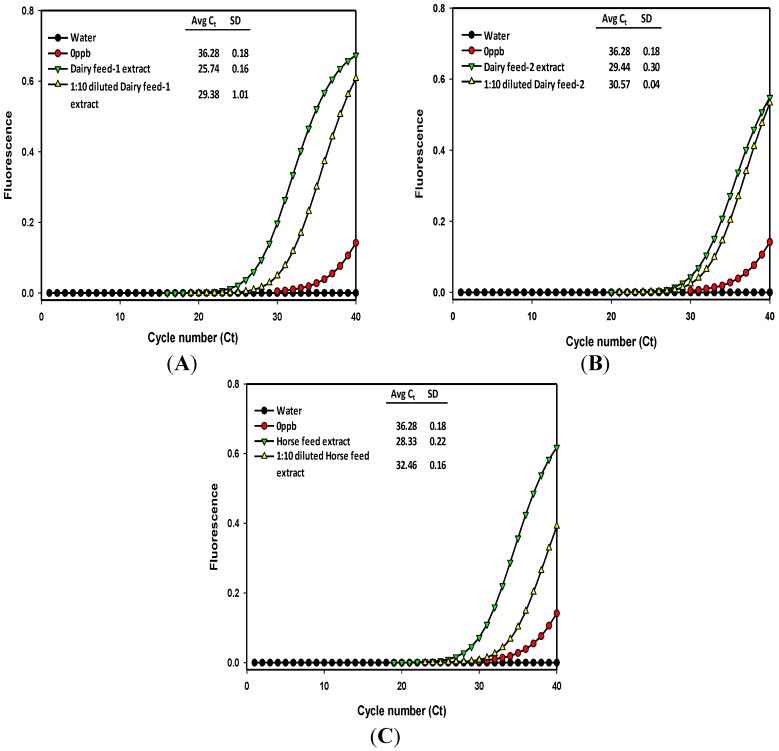
Comparison of immuno-PCR fluorescence signals obtained with dairy feed type 1 (**A**); dairy feed type 2 (**B**); and horse feed (**C**) extracts subjected to aflatoxin detection. Fluorescence signal curves were generated from the undiluted and 10-fold diluted methanol/water extracts. Legends indicate the average cycle threshold (*C_t_*) number and standard deviation from the replicates.

**Figure 3:**
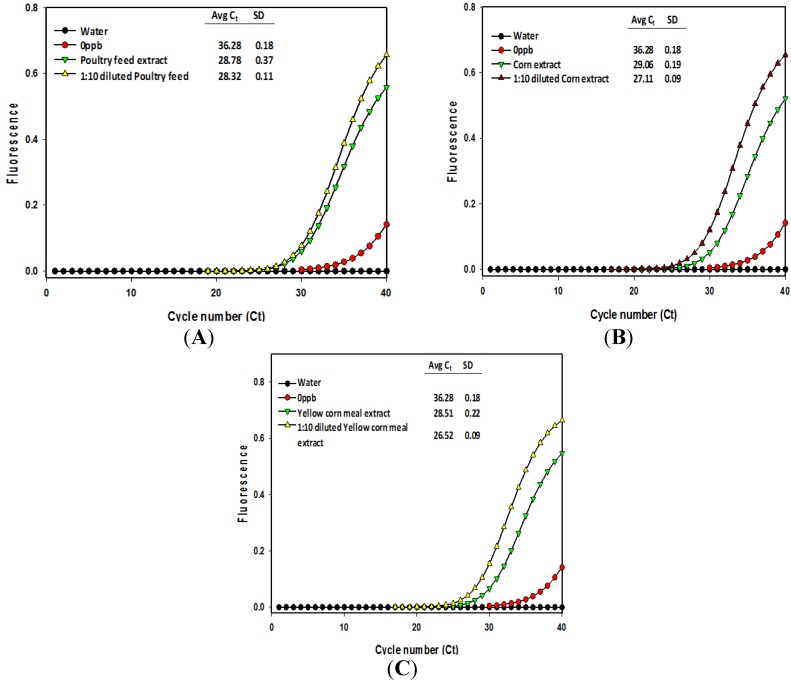
Comparison of immuno-PCR fluorescence signals obtained with poultry feed (**A**); corn (**B**); and yellow corn meal (**C**) extracts subjected to aflatoxin detection. Fluorescence signal curves were generated from the undiluted and 10-fold diluted methanol/water extracts. Legends indicate the average cycle threshold (*C_t_*) number and standard deviation from the replicates.

The undiluted methanol extracts of feeds, corn, and yellow corn meal samples were compared with respective 10-fold diluted extracts by subjecting them to immunomagnetic bead recovery of aflatoxin followed by real-time immuno-PCR quantification to generate the fluorescence signals [25]. In the case of dairy feed types 1 and 2, and horse feed extracts, the signals from the diluted extracts showed higher cycle threshold numbers and clear distinction from the signals of undiluted extracts (Figure 2 and Figure 3) meaning that 10-fold dilutions were reliable enough for quantification of these samples. Thus, the cycle threshold (*C_t_*) numbers from the signal plots of undiluted and 10-fold diluted extracts were used to calculate aflatoxin content using the linear regression equation of the calibration curve [25]. The aflatoxin content in undiluted extracts of dairy feed 1 and 2 and horse feed were estimated to be 4.18, 0.32, and 0.7 μg/kg, and for their 10-fold diluted extracts, the dilution corrected aflatoxin content was 3.38, 1.48, and 0.4 μg/kg, respectively (Table 2). Note that since the diluted extracts showed higher *Ct* values than the undiluted extracts, the aflatoxin content of 10-fold diluted extracts can be treated as the reliable estimate. However, in the case of poultry feed, corn grain, and yellow corn meal extracts, the *Ct* numbers of diluted extracts were lower than the *Ct* numbers of undiluted extracts, indicating excess antigen hook effects even after 10-fold dilution of the extracts (Figure 3). Although the diluted extracts of these samples showed aflatoxin content of 7.03, 16.23, and 24.40 μg/kg, respectively, further confirmation of aflatoxin capture and quantification may be obtained by using dilutions higher than 10-fold. Though, these samples were roughly estimated to contain aflatoxin amounts of around 20 μg/kg using the Agri-Screen test kit (Neogen, Lansing, MI, USA), this may also mean that the extraction efficiency of 60% methanol may be poor for poultry feed, corn grains, and yellow corn meal, which needs further attention.

**Figure 4:**
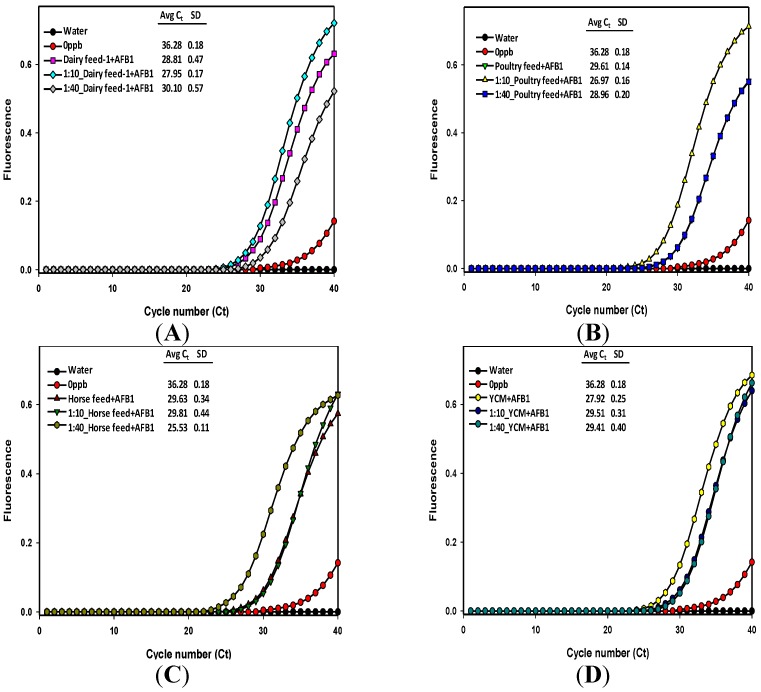
Comparison of immuno-PCR fluorescence signals from the 200 μg/kg AFB1 spiked dairy (**A**); poultry (**B**); horse feed (**C**); and yellow corn meal (**D**) extracts subjected to aflatoxin detection. Fluorescence signal curves were generated from the undiluted, 10- and 40-fold diluted methanol/water extracts. Legends indicate the average cycle threshold (*C_t_*) number and standard deviation from the replicates.

Testing for the validity and accuracy of our RT-iqPCR assay was also done by spiking the finely ground feed samples with a known concentration (200 μg/kg) of pure aflatoxin B_1_ standard prepared in methanol (Figure 4). The fluorescence signals that were generated, and their respective *Ct* numbers, are shown in Figure 4 and the calculated aflatoxin contents in Table 2. Note that for dairy feed 1, poultry feed, and yellow corn meal, the *Ct* numbers of diluted extracts are higher than the previous diluted or undiluted extracts. For spiked dairy feed, the *Ct* numbers and calculated aflatoxin content of the undiluted extract and the 40-fold diluted extracts were 28.81 (0.5 μg/kg) and 30.1 (8.21 μg/kg), respectively; for the spiked poultry feed, the *Ct* numbers of 10-fold diluted and 40-fold diluted extracts were 26.97 (17.88 μg/kg) and 28.96 (18.06 μg/kg), respectively, whereas for the spiked yellow corn meal, the *Ct* numbers and calculated aflatoxin content of undiluted extracts and the 10- and 40-fold diluted extracts were 27.92 (0.93 μg/kg), 29.51 (3.09 μg/kg), and 29.41 (13.23 μg/kg), respectively. In the case of the spiked horse feed extracts, the fluorescence plots indicated an aflatoxin content of 193.5 μg/kg in the 40-fold diluted extracts; this overestimation could be due to high antigen hook effect and may not be due to efficient recovery of added aflatoxin because the cycle threshold number of 25.53 was lower than the 10-fold (29.81) and undiluted (29.63) extracts. The *Ct* numbers for certain diluted extracts are generally higher than the undiluted or previous dilutions because the starting concentration of template DNA available for PCR is generally lowered due to dilution effect (reduced antigen concentration and reduced detection antibody-DNA), which resulted in signal detection at later cycles. Overall, the spiked feed and corn meal extracts did not show a good estimation and recovery of the added aflatoxin in our assay. This could be due to the complex nature of feed samples and the dependency of the aflatoxin recovery on the solvent chosen for extraction. The efficiency of aflatoxin extraction is known to be concentration-dependent, suggesting that the recovery of aflatoxin is better when the native aflatoxin concentration in a sample is at low levels than for samples at higher concentrations [34]. This indicated that the spiking levels and the efficiency of the extraction solutions should be pre-determined for a particular feed sample before subjecting to routine immuno-PCR assays. In addition, comparing with other conventional methods like ELISA and HPLC can further help to test the effect of inherent composition of different feed samples affecting the final results. However, these methods rely heavily on sample cleanup and by demonstrating that our approach does not need any sample cleanup, we are emphasizing the application of the immuno-PCR method. Comparing with other methods would also provide relative information on cost, time, sample size *etc.*, but we did not place emphasis on these topics in this study.

**Table 2:** Real-time iq-PCR quantification of aflatoxin in animal feeds, corn feed grain, and yellow corn meal samples. The calculated and dilution-corrected aflatoxin (μg/kg) concentrations are shown for the undiluted and diluted methanol/water extracts of un-spiked and spiked samples.

Methanol/water (60:40) extracts	Cycle threshold (*C_t_*) number ^1^	Calculated aflatoxin (μg/kg)	Dilution-corrected aflatoxin (μg/kg)
Dairy feed 1	25.74 ^a^	4.18	
Dairy feed 1 (1:10)	29.38 ^b^	0.34	3.38
Dairy feed 2	29.44 ^a^	0.32	
Dairy feed 2 (1:10)	30.57 ^b^	0.15	1.48
Horse feed	28.33 ^a^	0.70	
Horse feed (1:10)	32.46 ^b^	0.04	0.40
Poultry feed	28.78 ^a^	0.51	
Poultry feed (1:10)	28.32 ^a^	0.70	7.03
Corn feed grains	29.06 ^a^	0.42	
Corn feed grains (1:10)	27.11 ^b^	1.62	16.23
Yellow corn meal	28.51 ^a^	0.62	
Yellow corn meal (1:10)	26.52 ^b^	2.44	24.40
Dairy feed 1 + AFB1	28.81 ^a^	0.50	
Dairy feed 1 + AFB1 (1:10)	27.95 ^b^	0.91	9.08
Dairy feed 1 + AFB1 (1:40)	30.1 ^c^	0.21	8.21
Poultry feed + AFB1	29.61 ^a^	0.29	
Poultry feed + AFB1 (1:10)	26.97 ^b^	1.79	17.88
Poultry feed + AFB1 (1:40)	28.96 ^c^	0.45	18.06
Horse feed + AFB1	29.63 ^a^	0.28	
Horse feed + AFB1 (1:10)	29.81 ^a^	0.25	2.51
Horse feed + AFB1 (1:40)	25.53 ^b^	4.84	193.53
Yellow corn meal + AFB1	27.92 ^a^	0.93	
Yellow corn meal + AFB1 (1:10)	29.51 ^b^	0.31	3.09
Yellow corn meal + AFB1 (1:40)	29.41 ^b^	0.33	13.23

^1^ Data within the same feed set with different lower case letters are significantly different (*p* < 0.05).

## 3. Experimental Section

### 3.1. Animal Feeds, Feed Grain, and Yellow Corn Meal Samples

Four finished animal feed samples were obtained from the Willard Sparks Cattle Research Center, Animal Science Department of Oklahoma State University. Five-pound samples of various types of animal feed (poultry feed, dairy feed types 1 and 2, horse feed, and a whole kernel corn feed grain sample) were collected directly from the feed storage facility. The animal feeds selected contained the most commonly used ingredients in the respective animal diets. The samples were properly mixed in a large container and ground to fine powder using a household electric coffee grinder (capable of finely grinding coffee beans to less than 300-micron size) and stored at 4 °C. A finely ground food sample of yellow corn meal was also obtained from a local grocery store.

### 3.2. Anti-Aflatoxin Antibodies and Magnetic Beads

Anti-aflatoxin B_1_ polyclonal antibody raised in rabbit (part#A-8679) was supplied by Sigma-Aldrich (St. Louis, MO, USA), and monoclonal anti-aflatoxin B_1_ (AFC-13 IgG1 isotype) produced in mouse (part# sc-69863) was purchased from Santa Cruz Biotechnology Inc. (Santa Cruz, CA, USA). PureProteome™ Protein G magnetic beads (part# LSKMAGG02) were purchased from Millipore (Billerica, MA, USA) and used as a solid support for sandwich immunoassays. The magnetic beads were pre-washed and resuspended in a citric acid buffer (pH 5.0) containing 4.7 g/L citric acid and 9.2 g/L dibasic sodium phosphate (Na_2_HPO_4_) dehydrate. The blocking step of the beads involved use of normal rabbit serum (part# 011-000-001) supplied by Jackson Immuno-Research Laboratories Inc. (West Grove, PA, USA).

### 3.3. Aflatoxin Extraction

Aflatoxin B_1_ analytical standard was purchased from Supelco Analytical (Bellefonte, PA, USA), containing 20 μg AFB1/mL in 100% methanol. Feed and grain samples were finely ground using a household electric coffee grinder and the ground samples (<300 microns) were subjected to solvent extraction as follows. A 50-g ground sample was mixed with 100 mL of 60:40 HPLC grade methanol/water solvent in a screw cap tube and left for 30 min at room temperature with vigorous shaking. After centrifugation at 3000 rpm for 15 s, the supernatant was collected in a separate tube. Sample extractions were stored at 4 °C and subjected to magnetic bead recovery of aflatoxin on the same day, as explained below.

### 3.4. Aflatoxin Detection Using CD-ELISA

An Agri-Screen aflatoxin test kit (Neogen Corporation, Lansing, MI, USA, part#8010) that provides a visual semi-quantitative estimation of total aflatoxins in methanol extracts based on competitive direct enzyme-linked immunosorbent assay (CD-ELISA) was used for comparison. The Agri-Screen test kit (Neogen, Lansing, MI, USA) has been approved by the USDA’s Grain Inspection, Packers and Stockyards Administration (USDA/GIPSA #2006-09) and the Association of Analytical Communities (AOAC official method #990.32) and is widely used by quality control personnel worldwide for several commodities including corn, peanuts, feed grains, and mixed feeds. Using this kit, the methanol extracts of mixed feeds, corn, and yellow corn meal were screened for aflatoxin against a known control concentration of 20 μg/kg aflatoxin, as recommended by the manufacturer. Briefly, a 100 μL aliquot of the thoroughly mixed sample extract solution (60:40 methanol/water) was combined with an equal volume of enzyme-conjugated aflatoxin in a mixing well and added on to anti-aflatoxin antibodies immobilized in a microwell. The competitive reaction for the available antibody binding sites was allowed for two minutes between the free toxin in the sample (or control) and the added enzyme-conjugated toxin. The wells were washed five times with deionized water and, after the washing step, a 100 μL aliquot of substrate was added that reacts with the bound enzyme conjugate to produce a blue color. After 3 min, 100 μL of stop solution was added to end the reaction and the resultant color of the sample and control were visually compared, as recommended by the manufacturer.

### 3.5. Spiking of Feed and Food Samples

A 50-g sample of finely ground feed/food was spiked by adding 10 mL of 200 μg/kg of aflatoxin B_1_ prepared in 60% methanol. After mixing vigorously, the spiked samples were stabilized at room temperature for 30 min. Extractions of spiked samples were prepared as described above, using 90 mL of 60:40 methanol/water solvent to collect the clear supernatant. The extracted samples were stored at 4 °C and subjected to magnetic bead recovery of aflatoxin on the same day, as explained below. The dilution protocol strategy for the spiked samples was used to fit the aflatoxin estimation under the detection limits of the immuno-PCR assay and to avoid false negative results due to excess antigen hook effect. The aflatoxin wastes, toxin-spiked food/feed, and toxin-positive grain/feed samples were detoxified or decontaminated with bleach according to the USDA guidelines prior to autoclaving and disposal as biohazardous wastes.

### 3.6. Preparation of Reporter DNA and Detection Antibodies

The signal-generating complex involved capturing of aflatoxin between a capture antibody and a reporter DNA-conjugated detection antibody in a noncompetitive sandwich immune-quantitative PCR (RT-iqPCR). The reporter DNA marker was generated using the modified protocol of Wu and co-workers [35], as described in [25]. Briefly, a 563-bp fragment of firefly (*Photinus pyralis*) *luciferase* gene was amplified from the pGL2 plasmid vector (Catalog# E1641, Promega, Madison, WI, USA) using a 5'-C_6_ amino-modified forward primer (pGL2A_Fwd), as shown in Table 1. The reporter DNA with 5'-NH_2_ group was chemically tethered to an anti-aflatoxin polyclonal antibody using a heterobifunctional cross linker, sulfosuccinimidyl-4-(*N*-maleimidomethyl) cyclohexane-L-carboxylate (Sulfo-SMCC, catalog # 22622, Thermo Fisher Scientific Inc., Rockford, IL, USA), and this antibody-DNA conjugate was used as the secondary detector antibody.

### 3.7. Sandwich Immunoassay and Immunomagnetic Bead Recovery of Aflatoxin from Sample Extracts

Immunomagnetic bead recovery of aflatoxin from methanol/water extractions of animal feeds, corn, and yellow corn meal was done at room temperature by capturing with 1 μg (5 μL) of AFC-13 monoclonal anti-AFB1 antibodies in a 1.5 mL microcentrifuge tube containing 100 μL of extract. After a short (5 min toxin capture, 10 μL of PureProteome™ protein G magnetic beads (pre-washed in a citrate-phosphate wash buffer, pH 5.0) were added to immobilize the toxin-captured antibodies at room temperature with gentle shaking to avoid settling of the beads. After 15 min incubation, the immunomagnetic beads were allowed to form a pellet by using a magnet and were washed once with 200 μL of the citrate-phosphate wash buffer. A 25% normal rabbit serum (Jackson ImmunoResearch Laboratories Inc., West Grove, PA, USA) diluted in the wash buffer was used to block the beads for 5 min before adding 100 μL of detection antibody (reporter DNA-conjugated polyclonal anti-AFB1 antibody containing 0.0075 ng DNA/μL). A short incubation time of 5 min was employed for formation of the sandwich, avoiding nonspecific binding of the detection antibody, which occurs when prolonged incubation is done with the detection antibodies. Immediate recovery of the magnetic beads supporting the complex of ‘mouse anti-AFB1 monoclonal antibody-aflatoxin B_1_-rabbit anti-AFB1 polyclonal antibody’ sandwich was done by magnetic retention. Washing of the complex was done twice, each time with 200 μL citrate-phosphate wash buffer followed by washing twice with nuclease-free water. The beads were further collected in 100 μL nuclease-free water and heated for 10 min at 80 °C in 0.2 mL PCR tubes to release the bound molecules. After brief centrifugation at 5000 RPM, the supernatant was collected in new tubes and used for real-time PCR detection.

### 3.8. Dilution Protocols to Overcome Hook Effect

As previously demonstrated in our earlier paper [25], we suggested the use of dilution protocols to avoid the hook effect occurring in the immune assay because of excess antigen, and to reduce nonspecific binding of detection antibodies. Methanol extractions of the animal feed and feed grain samples were subjected to toxin capture as both undiluted and diluted aliquots were prepared at 1:4, 1:10, and 1:40 dilutions in distilled water. This dilution protocol was adapted to test the possibility of obtaining a fluorescence signal curve in RT-iqPCR that comes later (with a higher cycle number) than the signal from respective undiluted or lower dilution during the PCR amplification, indicating a weaker signal response concomitant with a lower sample amount due to dilution. This would help with verifying the method and quantifying toxin levels beyond the detection limits of the assay.

### 3.9. Detection and Quantification of Aflatoxin B_1_ Using Real-Time Immunoquantitative-PCR (RT-iqPCR)

Development of the RT-iqPCR assay for aflatoxin detection and quantification in animal feeds and feed grains involved initial optimization and troubleshooting mainly aimed at sensitive detection, signal amplification, and minimizing nonspecific binding of DNA-conjugated secondary detection antibodies. At first, the real-time PCR reaction itself was optimized with gradient concentrations of forward and reverse primers (Table 1), amplifying an internal fragment of the reporter DNA in order to obtain higher signals in positive samples and to see the absence of fluorescence signals in negative controls (nuclease-free water). A combination of 80-nM concentration of primers and the PerfeCTa^®^ SYBR^®^ Green I FastMix^®^ proved to give better amplification efficiency (99.5%) and fast cycling (data not shown). The reaction efficiency was tested to meet quantification requirements of real-time PCR using dilutions of antibody-conjugated reporter DNA and each iPCR reaction included melting curve analyses to detect nonspecific products, if any. A standard calibration curve using real-time immune-quantitative PCR was developed and the aflatoxin detection and quantification in animal feeds and food samples were performed using the optimized calibration curve, as we have described earlier [25]. Briefly, a 101-bp internal region of the reporter DNA was amplified in a 20 μL reaction mix containing 1× concentration of PerfeCTa^®^ SYBR^®^ Green I FastMix^®^ (Quanta BioSciences Inc., Gaithersburg, MD, USA) and 80 nM concentrations of pGL2B primers (Table 1). During the PCR amplification of reporter DNA, the increase in fluorescence signals after each PCR cycle was recorded by the Opticon-2 software to arrive at a threshold cycle value (*C_t_* number, the cycle number where the fluorescence signal crosses a manually set threshold, showing linear signal increase). The obtained *C_t_* values were used to inversely correlate with antigen concentrations, wherein the ‘no template control’ or ‘antigen negative controls’ would have the highest numerical *C_t_*, which decreases with increasing template concentrations. Using the calibration curve, the aflatoxin content of samples was estimated by correlating the *C_t_* value of the recovered DNA with the standard toxin concentrations (Figure 2). The quantification of aflatoxin in animal feeds, corn, and yellow corn meal samples was done directly by using their methanol extracts and also by using the extracts of samples spiked with 200 μg/kg of aflatoxin B_1_, as described by Babu and Muriana [25]. Briefly, the methanol/water extracts of each sample were individually subjected to magnetic bead recovery of aflatoxin, wherein a capture monoclonal antibody bound to the magnetic beads was used to recover the aflatoxin from the extract and then sandwiched between DNA-tethered detection antibodies. The complex was washed and subjected to RT-iqPCR quantification.

### 3.10. Statistical Analysis

Statistical analysis of fluorescence signals and estimation of aflatoxin content in unknown samples was done using SigmaPlot (ver. 11, Systat, San Jose, CA, USA) software for linear regression analysis of the four intra-assay replicates. Real-time PCR efficiency was used in plotting the standard curves. Standard deviation values for cycle threshold numbers (*C_t_*) were obtained using Opticon-2 software, which computes the population standard deviation evaluated over the entire set of sample *C_t_* values. Similarly, the aflatoxin contents (μg/kg) were calculated based on the linear regression equation, and population standard deviations were calculated using MS Excel software (Microsoft, Redmond, WA, USA). One-way analysis of variance (ANOVA) was used to determine significant differences, with an overall significance level of 0.05; pairwise comparisons were completed using the Holm-Sidak method within the statistics analysis functions in SigmaPlot (Systat, San Jose, CA, USA).

## 4. Conclusions

Developing rapid, sensitive, and specific analytical methods for detecting and quantifying aflatoxins can effectively address sub-chronic toxicity issues associated with food and feedstuffs containing trace levels of aflatoxin. The real-time immune-quantitative PCR (RT iq-PCR) method discussed in this study is able to detect aflatoxin levels lower than 20 ug/kg in feeds and feedstuffs, and also detects the high antigen hook effects commonly encountered in immunoassays. Although this method does not need sample cleanup, we recommend using dilution strategies for estimating potential problems with excess aflatoxin content in complex matrices of food and feed extracts. The recovery of high aflatoxin levels from spiked samples seemed to be poor using the 60% methanol solvent and needs further study. Where many samples of the same food/feed are to be tested, it may be prudent to perform standard curves with the same food/feed type in order to obtain similar extraction efficiencies with experimental samples.
